# MFG-E8 inhibits neutrophil migration through α_v_β_3_-integrin-dependent MAP kinase activation

**DOI:** 10.3892/ijmm.2015.2196

**Published:** 2015-04-23

**Authors:** MONOWAR AZIZ, WENG-LANG YANG, LANA M CORBO, WAYNE W CHAUNG, SHINGO MATSUO, PING WANG

**Affiliations:** Center for Translational Research, The Feinstein Institute for Medical Research and Department of Surgery, Hofstra North Shore-LIJ School of Medicine, Manhasset, NY, USA

**Keywords:** milk fat globule-epidermal growth factor-factor 8, neutrophil, HL-60 cells, MAP kinase, p38, ERK, G protein-coupled receptor kinase 2, CXCR2

## Abstract

We have previously demonstrated the involvement of milk fat globule-epidermal growth factor-factor 8 (MFG-E8) in reducing neutrophil infiltration in a murine model of acute lung injury (ALI). In the present study, we aimed to delineate the mechanisms through which MFG-E8 attenuates neutrophil migration. Recombinant human MFG-E8 (rhMFG-E8) was expressed and purified in our facility. The human differentiated neutrophil cell line, dHL-60, was treated with rhMFG-E8 and cell migration assay was performed in a Boyden chamber using recombinant interleukin-8 (IL-8) as the chemoattractant. Surface CXCR2 and intracellular G protein-coupled receptor kinase 2 (GRK2) levels were evaluated by flow cytometry or western blot analysis. The levels of mitogen-activated protein (MAP) kinases were determined by western blot analysis. Treatment with rhMFG-E8 resulted in a significant inhibition of dHL-60 cell migration in a dose-dependent manner. There was a 46% decrease in CXCR2 expression in the rhMFG-E8-treated dHL-60 cells, which was associated with a 32% increase in GRK2 expression. In the dHL-60 cells, treatment with rhMFG-E8 promoted the phosphorylation of p38 and extracellular signal-regulated kinase (ERK) within 10–30 min. The use of SB203580, a p38 inhibitor, and PD98059, an ERK inhibitor, resulted in the restoration of dHL-60 cell migration which was significantly inhibited treatment with rhMFG-E8. Furthermore, blocking the MFG-E8 receptors, α_v_β_3_/α_v_β_5_-integrins, by anti-α_v_-integrin neutralizing antibody (Ab) inhibited the activation of p38 and ERK, and reversed the rhMFG-E8-induced inhibition of dHL-60 cell migration. Finally, treatment of the dHL-60 cells with SB203580 and PD98059 neutralized the rhMFG-E8-induced downregulation of CXCR2 expression and upregulation of GRK2 expression, as well as the inhibitory effects on cell migration. Our findings reveal a novel mechanism of action of MFG-E8 through which it inhibits neutrophil migration through α_v_β_3_-integrin-dependent MAP kinase activation.

## Introduction

Neutrophils are the first cell type recruited to infected tissue; they sterilize the wound, clearing out invading bacteria through phagocytosis and subsequent killing by the release of reactive oxygen species (ROS) (1,2). Activated neutrophils also secrete numerous cytokines, chemokines, proteolytic enzymes stored in preformed granules, and pro-inflammatory products of arachidonic acid (prostaglandin E2 and leukotrienes), which collectively serve to recruit additional immune cells, remove cell debris and fine-tune the adaptive immune response (1,2). Although these functions of neutrophils are crucial components of normal wound healing, exaggerated or long-term neutrophil activity can contribute to tissue damage as a result of the uncontrolled release of ROS into the extracellular milieu (1,2). Under normal conditions, resting neutrophils have a short half-life and undergo apoptosis in the circulation after 6–9 h (3). However, when neutrophils reach a site of inflammation, apoptosis is delayed by inflammatory cytokines present in the tissues, which not only provide additional time for completion of their microbicidal function, but also exaggerate inflammation and tissue injury (4). Thus, neutrophils function as a ‘double-edged sword’. In order to prevent these adverse effects due to the release of proteolytic enzymes, such as elastase and myeloperoxidase and ROS, neutrophils should either be removed quickly from the inflamed tissue or their recruitment should be tightly controlled. Neutrophil recruitment is critical to pulmonary inflammatory responses associated with acute lung injury (ALI) (5,6). In our previous studies utilizing animal models of sepsis, ALI, and gut ishchemia-reperfusion (I/R) we observed an enhanced neutrophil infiltration at multiple organs, particularly into the lungs, liver and intestines, which led to the disruption of endothelial barrier function and promoted extravascular host tissue damage during uncontrolled inflammation (7-9). Conversely, the use of therapeutic regimens that restrict tissue neutrophil infiltration may help the host to overcome serious diseases in which abnormal neutrophil infiltration is a major concern.

Neutrophil trafficking into pulmonary tissue and air spaces in response to a gradient of chemoattractant is essential for their localization at sites of infection and inflammation to execute their functions (1). Neutrophil migration into the lungs is mediated by a complex cascade of rolling, adhesion and transendothelial migration, involving a number of factors that have already been well defined (10). Neutrophil infiltration into the lungs is mediated by a local production of chemokines released by macrophages, as well as other cell types in response to inflammation (11,12). The levels of CXC chemokines, such as interleukin-8 (IL-8) are significantly elevated in the bronchoalveolar lavage fluid (BALF) of patients with acute respiratory distress syndrome (ARDS), and increased IL-8 levels have been shown to be associated with increased neutrophil infiltration (13,14). In rodents, the IL-8 homologue, CINC-1/2, and MIP-2 regulate neutrophil recruitment into the lungs during experimental ALI through the chemokine receptor, CXCR2 (15,16). CXCR2 is a member of the G protein-coupled receptor (GPCR) superfamily and is expressed in neutrophils, monocytes and T cells (17). CXCR2 mediates neutrophil chemotaxis in response to tissue injury and many types of infections (17,18). G protein-coupled receptor kinase 2 (GRK2) has emerged as a key regulator of GPCR and other plasma membrane receptors triggered by chemotactic messengers (19). It has been demonstrated that the expression, localization and function of CXCR2 in polymorphonuclear leukocytes (PMNs) are tightly regulated by intracellular GRK2 (20–22). Upon activation, GRK2 phosphorylates CXCR2 and causes receptor desensitization and internalization, leading to the downregulation of neutrophil chemotaxis (20–22). Increasing evidence points to the occurrence of complex mechanisms modulating the subcellular localization, activity and expression levels of GRK2, which reveals new functional interactions of this kinase with various cellular proteins and transduction cascades (23). It has also been reported that GRK2 co-localizes with the mitogen-activated protein (MAP) kinases, and its activity and bidirectional regulation are mediated through the MAP kinase pathways (23–25).

Milk fat globule-epidermal growth factor-factor 8 (MFG-E8) is a secretory glycoprotein with bivalent binding activity to α_v_β_3_-integrin and acidic phospholipids, such as phosphatidylserine (PS) capable of carrying out versatile functions in cell physiology, such as the recognition of target cells and membrane vesicles by phagocytes (26,27), the development of male reproductive organs and cells (28), cell reorganization in mammary gland development and involution (29,30), and the regulation of inflammatory responses, such as macrophage activation (31,32) and neutrophil infiltration (8). It was named after its origin and structural properties, i.e., its origin in milk fat globule and its sequence homology to epidermal growth factor (EGF)-like domains of *Drosophila* Notch protein and C-terminal domains of human coagulation factors VIII and V (F5/8-type C domain). In our previous studies, we observed a significant decrease in MFG-E8 expression in the immune reactive organs following sepsis, ALI and gut I/R injury, and exogenous treatment with recombinant murine MFG-E8 (rmMFG-E8) markedly improved survival by attenuating systemic inflammation and the infiltration of neutrophils at vital organs (8,9,33). Therefore, in the present study, we aimed to elucidate the pivotal mechanisms through which MFG-E8 regulates neutrophil migration in response to the chemoattactant, IL-8. Based on our hypothesis, we demonstrate that the treatment of the human neutrophil-like cell line, HL-60, with recombinant human MFG-E8 (rhMFG-E8) results in a decreased migration ability towards IL-8. We further clarified the pivotal role of MFG-E8 in the α_v_β_3_-integrin mediated downregulation of neutrophil migration by modulating the surface expression of CXCR2 through GRK2-dependent pathways. We also deduced a novel and previously unexplored mechanism involving the MAP kinase pathways in the effects of MFG-E8 on the inhibition of neutrophil migration. Importantly, the present findings identify an additional role of MFG-E8 in inhibiting neutrophil infiltration through MAP kinase-dependent pathways. Thus, this may prove to be an effective therapeutic strategy in the treament of diseases in which enhanced neutrophil infiltration is a major concern.

## Materials and methods

### HL-60 cell culture and differentiation

HL-60 human promyelocytic leukemia cells, obtained from the American Type Culture Collection (ATCC; Manassas, VA, USA) were cultured in a T-25 cell culture flask at a density of 2×10^5^ cells/ml in 15 ml RPMI-1640 medium supplemented with 10% fetal bovine serum (FBS), and penicillin and streptomycin. The cells were kept in 37°C incubator under humidified conditions containing 5% CO_2_. The cells were grown to a density of 1×10^6^ cells/ml, at which time they were passaged by seeding into a new flask at 2×10^5^ cells/ml. In order to induce the differentiation of the HL-60 [differentiated HL-60 (dHL-60)] cells, 1×10^5^ cells/ml at the mid-log growth phase were grown in a T-25 flask in 15 ml of RPMI-1640 medium containing 190 *µ*l of 100% dimethyl sulfoxide (DMSO) supplemented with 10% FBS, and penicillin/streptomycin antibiotics for a period of 5-7 days, as previously described (34), which induced their differentiation into PMNs.

### Stimulation of dHL-60 cells with rhMFG-E8

The expression, purification and functional characterization of rhMFG-E8 were performed according to a previously described protocol (35). In brief, a 1,095 bp fragment encoding the mature region of human MFG-E8 was obtained by polymerase chain reaction amplification and cloned into the *Sal*I and *Not*I site of the pET-28a(+) vector (Novagen, Inc., Madison, WI, USA) downstream of the phage T7 RNA polymerase promoter. The plasmid was transformed into *E. coli* BL21 (DE3) cells grown at 37°C in 2YT medium (Invitrogen Life Technologies, Grand Island, NY, USA) with kanamycin overnight. rhMFG-E8 protein production was induced by the addition of isopropyl-β-D-thiogalactopyranoside (IPTG) to a final concentration of 1.0 mM and cell growth continued for 5 h at 25°C. The cells were harvested by centrifugation at 6,000 rpm and the induced rhMFG-E8 protein was purified according to the manufacturer’s instructions (Novagen, Inc.). The rhMFG-E8 fractions were pooled and the endotoxin of the protein solution was removed by phase separation using Triton X-114. The content of lipopolysaccharide (LPS) in the sample was determined using the Limulus Amebocyte Lysate assay kit (BioWhittaker, Walkersville, MD, USA). The purity of rhMFG-E8 was evaluated by SDS-PAGE on a 10–20% Tris-HCl gel and visualized using the GelCode Blue Stain Reagent (Pierce Biotechnology, Inc., Rockford, IL, USA). The final product was concentrated by Amicon ultra-15 centrifugal filter devices to the designed concentration and stored at -20°C. For the stimulation of the dHL-60 cells with rhMFG-E8, a total of 1.5×10^6^ cells/ml was placed into 1.5 ml microcentrifuge tubes in serum-free Opti-MEM (Invitrogen Life Technologies) and then stimulated with rhMFG-E8 at a dose of 500 ng/ml for the indicated period of time. Subsequently, experiments were carried out for the assessment of cell migration, and intracellular signal transduction by western blot analysis and flow cytometry.

### In vitro cell migration assay

The migration assays were conducted in a modified 24-well (3.0 *µ*m) Boyden chamber (BD Biosciences, San Jose, CA, USA). Following differentiation, the dHL-60 cells (3×10^5^) were pre-treated with either rhMFG-E8 (125–1,000 ng/ml) or PBS for 2 h, and then plated in the Boyden chamber inserts and medium with 50 ng/ml of recombinant human IL-8 (rhIL-8; R&D Systems, Minneapolis, MN, USA) was placed in the outer compartment which served as a chemoattractant. After 1.5 h of incubation, the upper surface of the filter was swabbed with cotton-tipped applicators to remove non-migratory cells. The migrated cells were fixed with 4% paraformaldehyde (PFA) and stained with propidium iodide (PI) (1 *µ*g/ml). A total of 6 random microscopic fields per well were counted.

### Flow cytometric analysis

To examine the surface CXCR2 and intracellular GRK2 expression levels, the dHL-60 cells (1.5×10^6^ cells) treated with rhMFG-E8 (500 ng/ml) for 2 h were first surface-stained with PE-CXCR2 (BioLegend, San Diego, CA, USA), and subsequently, to determine intracellular GRK2 expression, the cells were fixed and permeabilized with IntraPrep (Beckman Coulter, Fullerton, CA, USA), followed by staining with FITC-GRK2 antibodies (Abcam, Cambridge, MA, USA). After washing, the stained cells were analyzed using a FACSVerse flow cytometer (BD Biosciences). Data were analyzed by FlowJo software (FlowJo, LLC, Ashland, OR, USA) with 15,000 events per sample. Isotype controls and Fc Receptor Blocker (both from BioLegend) were used for all the samples.

### Western blot analysis

The dHL-60 cells (1.5×10^6^/ml) were placed into 1.5 ml microfuge tubes with Opti-MEM and then stimulated with either rhMFG-E8 (500 ng/ml) or PBS for different periods of time. Following incubation, the cells were centrifuged at 200 x g for 5 min and the supernatants were removed. The cell pellet was then lysed by the addition of 80–100 *µ*l of loading buffer containing 0.5 M Tris-HCl, pH 6.8, 16% glycerol, 10% SDS and 1% Bromophenol Blue. The lysate was then heated to 95°C for 5 min and an equal volume (20 *µ*l) of each lysate per lane was loaded onto a 4–12% Bis-Tris gel (Invitrogen Life Technologies) and transferred onto a 0.2-*µ*m nitrocellulose membrane (Invitrogen Life Technologies). The membrane was incubated overnight at 4°C with the primary antibodies as obtained from respective vendors: rabbit anti-GRK2 monoclonal antibody (Cat. no. ab32558; Abcam), rabbit anti-phospho-p38 (Cat. no. 9211), anti-phospho-extracellular signal-regulated kinase (ERK)1/2 (Cat. no. 9101), and the total antibodies for p38 (Cat. no. 9212) and ERK1/2 (Cat. no. 4695), (all from Cell Signaling Technology, Danvers, MA, USA), at a 1:1,000 dilution, reacted with peroxidase-conjugated goat anti-rabbit secondary antibody (Cat. no. 4030-05; SouthernBiotech, Birmingham, AL, USA) at a 1:10,000 dilution at room temperature for 2 h, and washed 5 times in TBST. The immunoblot was washed, stripped off and reprobed with mouse anti-β-actin antibody (Cat. no. A2228; Sigma-Aldrich, St. Louis, MO, USA) as a loading control. The resulting signals were detected by ECL (GE Healthcare, Buckinghamshire, UK), and the band intensities were assessed by densitometry using ImageJ software, as previously described (36).

### In vitro neutralization of α_v_-integrin

For the *in vitro* neutralization of the α_v_-integrin receptor, a total of 1.5×10^6^ dHL-60 cells were placed into 1.5 ml microfuge tubes containing 1 ml of Opti-MEM. The cells were then pre-treated with 1 *µ*g/ml of each of the IgG isotype control or anti-α_v_-integrin neutralizing antibody (both from BioLegend) for 1 h at 37°C. Subsequently, the cells were stimulated with rhMFG-E8 (500 ng/ml) or PBS for different periods of time and then analyzed by flow cytometry or western blot analysis.

### Inhibition of p38 and ERK using chemical inhibitors

The dHL-60 cells were placed into 1.5 ml microfuge tubes at a density of 1.5×10^6^ cells/ml in Opti-MEM. The cells were then pre-treated with the p38 inhibitor, SB203580 and the ERK inhibitor, PD98059 (both from Tocris Bioscience, Ellisville, MO, USA), at a concentration of 10 *µ*M for each for 1 h at 37°C. Following incubation, the cells were stimulated with rhMFG-E8 or PBS for 2 h followed by the assessment of CXCR2 and GRK2 expression by flow cytometry and western blot analysis.

### Statistical analysis

All data are expressed as the means ± SE and compared by one-way ANOVA and the Student-Newman-Keul (SNK) test. The Student’s t-test was used when only 2 groups were compared. Differences in values were considered significant if P<0.05.

## Results

### rhMFG-E8 inhibits dHL-60 cell migration in a dose-dependent manner

**Figure 1:**
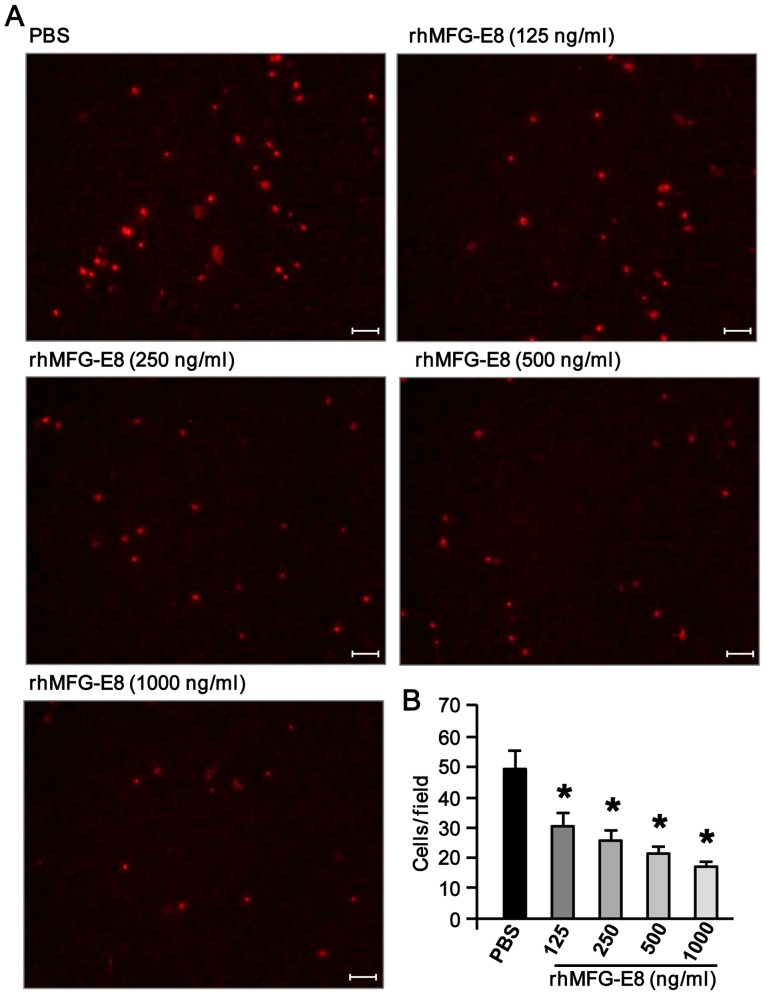
Recombinant human milk fat globule-epidermal growth factor-factor 8 (rhMFG-E8) attenuates HL-60 cell migration. (A) Differentiated HL-60 cells (3×10^5^) were pre-stimulated with different doses (1,000, 500, 250 and 125 ng/ml) of rhMFG-E8 or PBS for 2 h, and then plated in 500 *µ*l volume at the Boyden chamber inserts. The outer compartment of the inserts contained 500 *µ*l of RPMI medium with 50 ng/ml of recombinant human interleukin-8 (IL-8) as a chemoattractant. After 1.5 h of incubation, the upper surface of the filter was swabbed with cotton-tipped applicators to remove non-migratory cells. Migrated cells were fixed with 4% paraformaldehyde (PFA) and stained with propidium iodide (PI) (1 *µ*g/ml). A total of 6 random microscopic fields per well were counted. Scale bar, 100 *µ*m. (B) The average number of migrated dHL-60 cells is plotted in a bar diagram where the results are expressed as the means ± SE obtained from 6 fields/group of 3 independent experiments. ^*^P<0.05 vs. PBS treatment.

To examine the effects of rhMFG-E8 on neutrophil migration, the dHL-60 cells were pre-treated with various concentrations of rhMFG-E8 for 2 h. The cells were then allowed to proceed for migration towards rhIL-8 as a chemoattractant using a Boyden chamber. As shown in Fig. 1A and B, pre-treatment of the dHL-60 cells with rhMFG-E8 led to a significantly decrease in their migration towards rhIL-8 in a dose-dependent manner. Since the most notable decrease in their migration occurred following stimulation with rhMFG-E8 at the doses of 500 and 1,000 ng/ml, among these 2 doses we decided to utilize the lesser dose of 500 ng/ml of rhMFG-E8 protein for the subsequent *in vitro* experiments.

### rhMFG-E8 downregulates CXCR2 surface expression by upregulating intracellular GRK2 expression in dHL-60 cells

**Figure 2:**
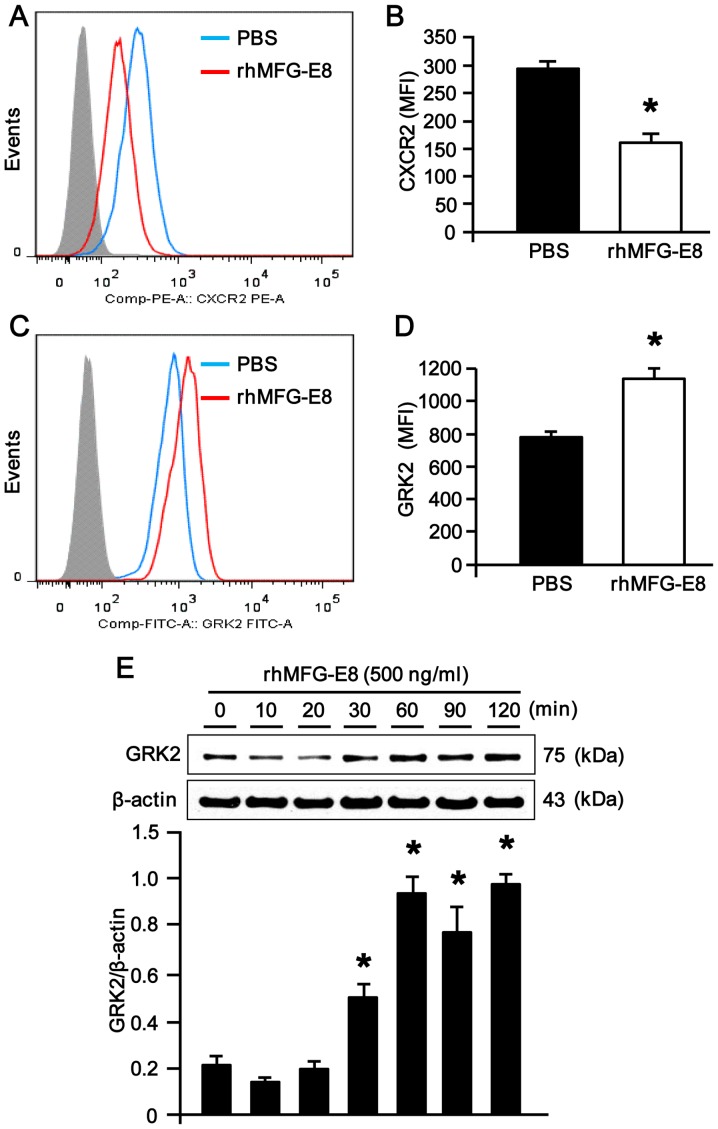
Expression of CXCR2 and G protein-coupled receptor kinase 2 (GRK2) in recombinant human milk fat globule-epidermal growth factor-factor 8 (rhMFG-E8)-treated neutrophils. (A) Differentiated HL-60 (dHL-60) cells (1.5×10^6^ cells) treated with rhMFG-E8 (500 ng/ml) for 2 h were surface-stained with PE-CXCR2 and then subjected to flow cytometric analysis. Data were analyzed by Flowjo software with 15,000 events per sample. Isotype controls and Fc receptor blocker were used for all the samples. The representative histograms for PBS and rhMFG-E8-treated dHL-60 cells obtained from 3 independent experiments are shown. (B) Bar diagram representing the mean fluorescence intensities (MFI) of the PBS- and rhMFG-E8-treated samples are shown. Data are expressed as the means ± SE (n=3 samples/group), obtained from 3 independent experiments. ^*^P<0.05 vs. PBS treatment. (C and D) To examine the expression of intracellular GRK2 levels, dHL-60 cells (5×10^6^ cells) treated with rhMFG-E8 (500 ng/ml) for 2 h were first surface-stained with PE-CXCR2, and then to examine intracellular GRK2 expression, cells were fixed and permeabilized with Intraprep, followed by staining with FITC-GRK2 antibodies. After washing, the stained cells were subjected to flow cytometry using a FACSVerse flow cytometer. Appropriate isotype controls and Fc receptor blocker were used for all the samples. Representative histogram and the bar diagrams indicating the MFI of PBS and rhMFG-E8-treated samples are shown. Data are expressed as the means ± SE (n=3 samples/group), obtained from 3 independent experiments. ^*^P<0.05 vs. PBS treatment. (E) Differentiated HL-60 cells (1.5×10^6^/ml) were placed into 1.5 ml microfuge tubes with Opti-MEM and then stimulated with either rhMFG-E8 (500 ng/ml) or PBS for different periods of time. Following incubation, the cell lysates were harvested and then subjected to western blot analysis using rabbit anti-GRK2 monoclonal antibody. Results were normalized to β-actin as an internal control and are expressed as the fold induction in comparison to the 0 min time point. Data are expressed as the means ± SE (n=3 samples/group), obtained from 3 independent experiments. ^*^P<0.05 vs. 0 min.

CXCR2, a surface receptor for the chemokine, IL-8, plays a crucial role in IL-8-dependent neutrophil migration (15,16,37). The dHL-60 cells stimulated with rhMFG-E8 showed a significant downregulation in CXCR2 expression at their cell surface (Fig. 2A and B), which was further linked to the decrease in neutrophil migration following stimulation with rhMFG-E8. Since intracellular GRK2 serves as a negative regulator of surface CXCR2 expression in neutrophils, we wished to assess intracellular GRK2 expression in the dHL-60 cells following stimulation with rhMFG-E8. Of note, we observed a significant upregulation in intracellular GRK2 expression in the dHL-60 cells stimulated with rhMFG-E8, as revealed by flow cytometric analysis (Fig. 2C and D). Consistently, western blot analysis of GRK2 protein expression also revealed the reproducible findings of its upregulation upon rhMFG-E8 stimulation in a time-dependent manner (Fig. 2E), suggesting that the inhibition of CXCR2 expression in dHL-60 cells may be mediated through the upregulation of GRK2 expression.

### rhMFG-E8 upregulates MAP kinase phosphorylation through α_v_β_3_-integrin

**Figure 3:**
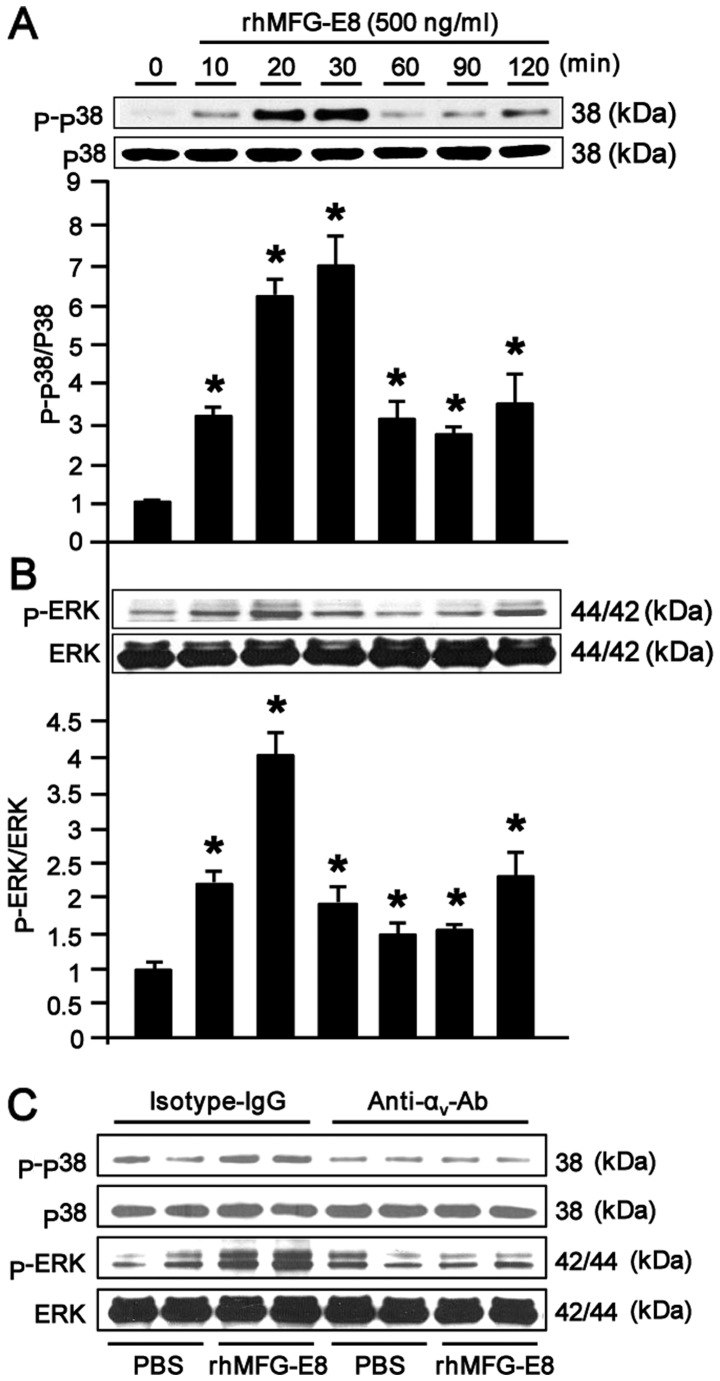
Recombinant human milk fat globule-epidermal growth factor-factor 8 (rhMFG-E8) activates mitogen-activated protein (MAP) kinases through α_v_β_3_-integrin. (A and B) Differentiated HL-60 cells (1.5×10^6^/ml) were placed into 1.5 ml microfuge tubes with Opti-MEM and then stimulated with rhMFG-E8 (500 ng/ml) for different periods of time. Following incubation, the cell lysates were harvested and subjected to western blot analysis using monoclonal antibodies for phospho and total p38 and ERK. Results are normalized with total p38 and ERK as loading control and are expressed as the fold induction in comparison to the 0 min time point. Data are expressed as the means ± SE (n=3 samples/group), obtained from 3 independent experiments.^*^P<0.05 vs. 0 min. (C) A total of 1.5×10^6^ dHL-60 cells was placed into 1.5 ml microfuge tubes containing 1 ml of Opti-MEM. The cells were then pre-treated with 1 *µ*g/ml of each of the IgG isotype control or anti-α_v_-integrin neutralizing antibody for 1 h at 37°C. The cells were then stimulated with rhMFG-E8 (500 ng/ml) or PBS for 30 min and then subjected to western blot analysis using antibodies for p-p38, and ERK, and total p38 and ERK. Representative blots obtained from 3 independent experiments are shown. ^*^P<0.05 vs. 0 min.

To determine whether rhMFG-E8 alters MAP kinase phosphorylation, the dHL-60 cells were treated with rhMFG-E8 for different periods of time and we then measured the p38 and ERK phosphorylation levels by western blot analysis. As shown in Fig. 3A and B, the dHL-60 cells stimulated with rhMFG-E8 showed a significant upregulation in p38 and ERK phosphorylation in a time-dependent manner with the highest induction in their phosphorylation observed at the 20- and 30-min time points; after these time points, their phosphorylation decreased to basal levels. Since α_v_β_3_-integrin recognizes MFG-E8, we wished to determine whether this integrin is involved in the MFG-E8-mediated signal transduction of p38 and ERK phosphorylation in the dHL-60 cells. For this purpose, we first treated the dHL-60 cells with the neutralizing antibody for α_v_-integrin or IgG isotype antibody followed by stimulation with rhMFG-E8, which evidently revealed that the promoting effects of rhMFG-E8 on p38 and ERK phosphorylation were notably diminished (Fig. 3C), indicating the role of α_v_β_3_-integrin in transducing MFG-E8-mediated downstream signaling and MAP kinase activation.

### rhMFG-E8 modulates CXCR2 and GRK2 expression through α_v_β_3_-integrin

**Figure 4:**
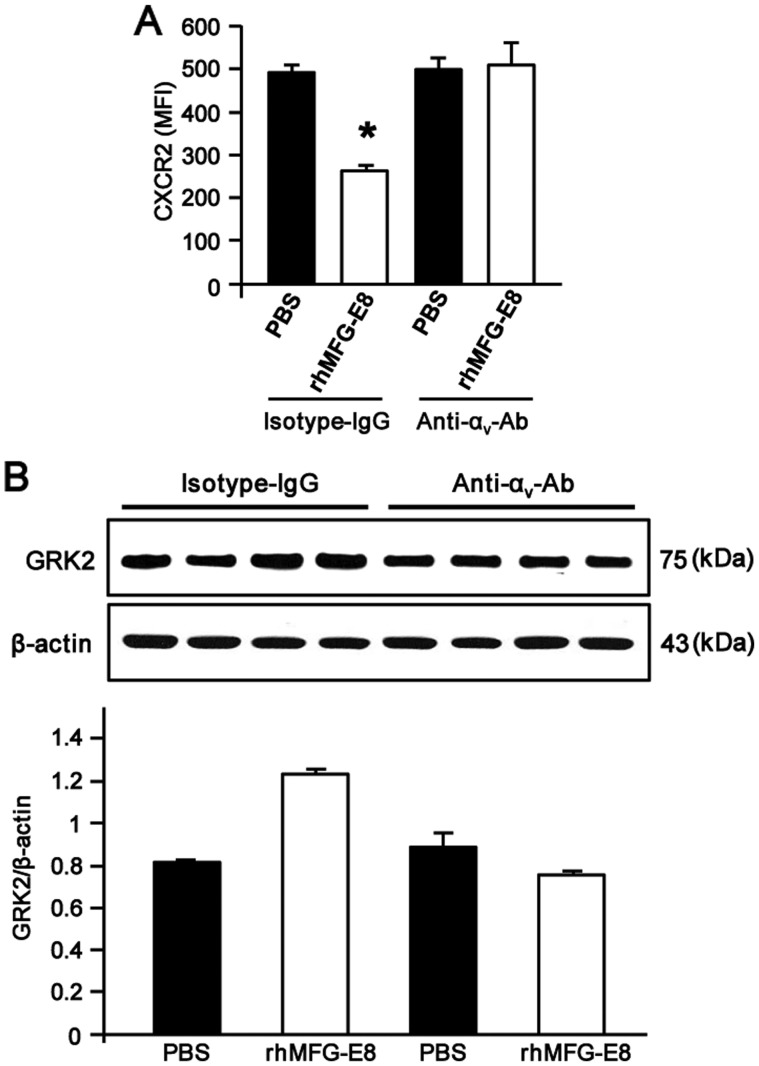
Recombinant human milk fat globule-epidermal growth factor-factor 8 (rhMFG-E8) regulates CXCR2 and G protein-coupled receptor kinase 2 (GRK2) expression through α_v_β_3_-integrin. (A) Differentiated HL-60 (dHL-60) cells (1.5×10^6^) were placed into 1.5 ml microfuge tubes containing 1 ml of Opti-MEM. The cells were pre-treated with 1 *µ*g/ml of each of the IgG isotype control or anti-α_v_-integrin neutralizing antibody for 1 h at 37°C. The cells were then stimulated with rhMFG-E8 (500 ng/ml) or PBS for 2 h and then subjected to flow cytometry using PE-labeled anti-CXCR2 antibody. The mean fluorescence intensities (MFI) of the isotype- and anti-α_v_-integrin-treated samples are shown. Data are expressed as the means ± SE (n=3 samples/group), obtained from 3 independent experiments. ^*^P<0.05 vs. PBS treatment. (B) A total of 1.5×10^6^ dHL-60 cells was placed into 1.5 ml microfuge tubes containing 1 ml of Opti-MEM. The cells were then pre-treated with 1 *µ*g/ml of each of the IgG isotype control or anti-α_v_-integrin neutralizing antibody for 1 h at 37°C. The cells were then stimulated with rhMFG-E8 (500 ng/ml) or PBS for 2 h and then subjected to western blot analysis using anti-GRK2 antibody. The blot was stripped off and re-probed for anti-β-actin antibody, which served as the loading control. Representative blots and corresponding densitometric bar diagram obtained from 2 independent experiments are shown.

To determine the involvement of α_v_β_3_-integrin in the rhMFG-E8-mediated alteration in CXCR2 and GRK2 expression, the dHL-60 cells were pre-treated with anti-α_v_-integrin antibody to block the MFG-E8 receptor for the transmission of downstream signaling. As shown in Fig. 4A, pre-treatment of the cells with anti-α_v_-integrin antibody neutralized the rhMFG-E8-induced downregulation in the surface expression of CXCR2, while a significant downregulation in CXCR2 surface expression was observed in the cells treated with the IgG isotype control. Similarly, the expression of GRK2 was induced in the rhMFG-E8-stimulated dHL-60 cells pre-treated with the IgG isotype control; conversely the rhMFG-E8-induced upregulation in GRK2 expression was diminished in the cells pre-treated with anti-α_v_-integrin (Fig. 4B). Taken together, these data clearly indicate that the MFG-E8-mediated down-regulation of CXCR2 and the upregulation of GRK2 expression are transmitted through the α_v_β_3_-integrin pathway.

### MAP kinase inhibitors neutralize the rhMFG-E8-induced inhibition of CXCR2 and enhancement of GRK2 expression

**Figure 5:**
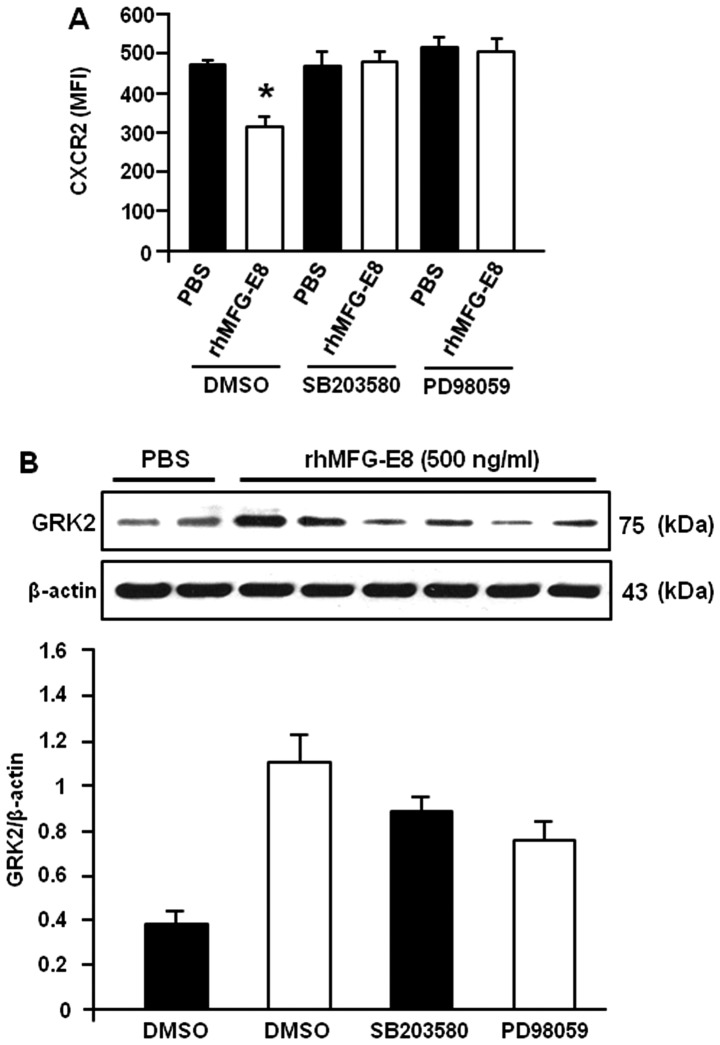
Recombinant human milk fat globule-epidermal growth factor-factor 8 (rhMFG-E8) regulates CXCR2 and G protein-coupled receptor kinase 2 (GRK2) expression through the p38 and ERK pathways. (A) Differentiated HL-60 (dHL-60) cells were placed into 1.5 ml microfuge tubes at a density of 1.5×10^6^ cells/ml of Opti-MEM. The cells were then pre-treated with the p38 inhibitor, SB203580, of the ERK inhibitor, PD98059, at a concentration of 10 *µ*M of each for 1 h at 37°C. Following incubation, the cells were then stimulated with rhMFG-E8 (500 ng/ml) or PBS for 2 h followed by the assessment of CXCR2 by flow cytometry. Mean fluorescence intensities (MFI) obtained from 3 independent experiments are plotted into the bar diagram.^*^P<0.05 vs. PBS treatment. (B) Western blot analysis using anti-GRK2 antibody. The blot was stripped off and re-probed for anti-β-actin antibody, which served as the loading control. Representative blots obtained from 2 independent experiments are shown. Densitometric data are expressed as the means ± SE (n=2 samples/group), obtained from 2 independent experiments.

To determine the role of MAP kinases in the rhMFG-E8-mediated inhibition of CXCR2 expression, the dHL-60 cells were pre-treated with MAP kinase inhibitors and the effects of rhMFG-E8 on CXCR2 expression were then evaluated. As shown in Fig. 5A, pre-treatment of the cells with the specific inhibitors of p38 and ERK diminished the negative regulatory effects of rhMFG-E8 on CXCR2 which led to the downregulation of its surface expression, indicating the involvement of MAP kinases in rhMFG-E8-mediated signaling. Similarly, the rhMFG-E8-induced upregulation of GRK2 was also diminished when the p38 and ERK molecules were blocked by using their specific inhibitors as compared to the DMSO control (Fig. 5B). These data clearly indicate that the rhMFG-E8-mediated downstream signaling which downregulates CXCR2 and upregulates GRK2 is mediated through MAP kinase activation.

### Inhibition of α_v_-integrin and MAP kinases diminishes the rhMFG-E8-mediated downregulation in neutrophil migration

**Figure 6:**
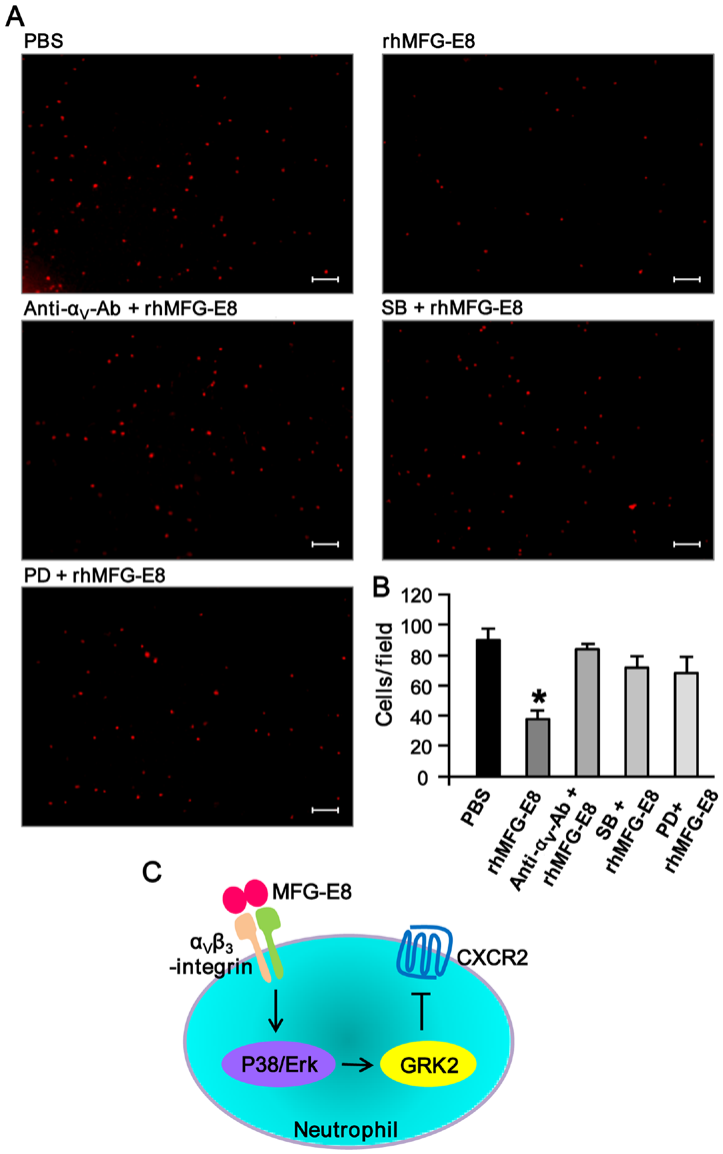
Treatment with anti-α_v_-integrin antibody and mitogen-activated protein (MAP) kinase inhibitors counteracts recombinant human milk fat globule-epidermal growth factor-factor 8 (rhMFG-E8)-mediated downregulation of neutrophil migration. (A) Differentiated HL-60 (dHL-60) cells (3×10^5^) were pre-stimulated with 1 *µ*g/ml of each of the IgG isotype control, anti-α_v_-integrin neutralizing antibody, the p38 inhibitor, SB203580 (SB), of the ERK inhibitor, PD98059 (PD), at a concentration of 10 *µ*M for 1 h at 37°C in their respective 1.5 ml microfuge tubes. The cells were then stimulated with rhMFG-E8 (500 ng/ml) or PBS for 2 h, and then plated in 500 *µ*l volume in the Boyden chamber inserts. The outer compartment of the inserts contained 500 *µ*l of RPMI medium with 50 ng/ml of recombinant human interleukin-8 (IL-8) as a chemoattractant. After 1.5 h of incubation, the upper surface of the filter was swabbed with cotton-tipped applicators to remove non-migratory cells. Migrated cells were fixed with 4% paraformaldehyde (PFA) and stained with propidium iodide (PI) (1 *µ*g/ml). A total of 6 random microscopic fields per well were counted. Scale bar, 100 *µ*m. (B) The average number of migrated dHL-60 cells are plotted in a bar diagram where the results are expressed as the means ± SE obtained from 6 fields/group of 3 independent experiments. ^*^P<0.05 vs. PBS. (C) Mechanistic finding. The secretory glycoprotein, MFG-E8 binds to its receptor, α_v_β_3_-integrin, and transduces downstream signaling of MAP kinase (p38 and ERK) activation. The activated MAP kinases then upregulate G protein-coupled receptor kinase 2 (GRK2) which in turn negatively regulates the surface exposure of CXCR2, thereby attenuating neutrophil migration.

In order to determine the involvement of α_v_β_3_-integrin and MAP kinases in the rhMFG-E8-mediated inhibition of neutrophil migration, the dHL-60 cells were pre-treated with anti-α_v_-integrin antibody and the MAP kinase inhibitors, and the effects of rhMFG-E8 on neutrophil migration were antibody evaluated. As shown in Fig. 6A and B, pre-treatment of the cells with anti-α_v_-integrin antibody and MAP kinase inhibitors markedly reversed the inhibitory effects of rhMFG-E8 on neutrophil migration. Collectively, these findings clearly indicate that MFG-E8 inhibits neutrophil migration by downregulating surface CXCR2 expression through the upregulation of the expression of the intracellular negative regulator, GRK2, and this event is mediated by α_v_β_3_-integrin-mediated MAP kinase activation (Fig. 6C).

## Discussion

Neutrophils are the first line of defense against tissue infection, trauma, stress insults and injury. These cells play a key role in the defense against bacterial, fungal and viral infections and a growing body of evidence suggests that neutrophils may also represent a critical link between the innate and adaptive immune system (1). Therefore, the migration of neutrophils to infected tissue and secondary lymphoid organs is critical for effective immune responses to the majority of pathogens; however, uncontrolled migration can lead to tissue damage and chronic inflammation (2). In our previous studies, we reported an enhanced infiltration of neutrophils into the vital organs following sepsis, ALI, renal, and gut I/R injury, causing severe inflammation and tissue damage. However, the deleterious events caused by excessive neutrophil accumulation were ameliorated by exogenous treatment with recombinant MFG-E8 which attenuated neutrophil migration and infiltration into tissues (8,9,38).

Although we have initially elucidated the involvement of the the downregulation of the IL-8 receptor, CXCR2, at the neutrophil cell surface due to the upregulation of its intracellular negative regulator, GRK2, in rmMFG-E8-treated murine bone marrow-derived neutrophils (8), in this study, in order to reveal the mechanisms through which MFG-E8 attenuates neurophil migration, we utilized the human neutrophil cell line, HL-60, and stimulated the cells with rhMFG-E8 and focused on evaluating the upstream signaling components which may result in the modulation of GRK2/CXCR2 signaling. We observed a marked induction in the levels of p-p38 and ERK MAP kinases following stimulation of the HL-60 cells with rhMFG-E8. To confirm this novel link between MFG-E8 and MAP kinases, we utilized a two-step blocking strategy: i) neutralization of the α_v_β_3_-integrin heterodimer by the anti-α_v_-integrin antibody abrogated the rhMFG-E8-induced increase in the levels of MAP kinases; and ii) inhibition of MAP kinases by their inhibitors diminished the inhibitory effects of rhMFG-E8 on neutrophil migration through the modulation of GRK2/CXCR2, thus suggesting the involvment of these two novel pathways in the MFG-E8-mediated downregulation of human neutrophil migration.

MFG-E8 has two functional domains: the N-terminal EGF domain that bind to α_v_β_3_-integrin of most hematopoietic cells, and the C-terminal discoidin domains that recognizes the PS in apoptotic cells (26). In this study, we considered α_v_β_3_-integrin as the gateway for MFG-E8-mediated signal transduction. Structurally, α_v_β_3_-integrin is a heterodimeric transmembrane receptor formed by the non-covalent association of the α and β subunits (39). In this study, we demonstrated that the blocking α_v_-integrin in neutrophils effectively diminished the effects of rmMFG-E8 on IL-8-mediated HL-60 cell migration through the activation of MAP kinases and the modulation of GRK2/CXCR2 expression, indicating the involvement of α_v_-integrin in mediating MFG-E8 activity. Similar to our results demonstrating that the blocking of α_v_-integrin abrogates MFG-E8 signaling, Cheyuo *et al* also adopted the same approach to block only α_v_-integrin for the functional assessment of MFG-E8-mediated anti-inflammatory and anti-apoptotic roles in cerebral ischemic injury (40). Furthermore, our findings identify the integrin signaling pathway as another critical factor in controlling GRK2-mediated CXCR2 downreg ulation.

It is already known that MFG-E8 was first discovered as a scavenging factor to promote the phagocytosis of apoptotic cells by macrophages through the formation of a bridge between them (26). However, MFG-E8 has several immunological and physiological functions. Apart from participating in the phagocytic clearance of apoptotic cells, MFG-E8 directly attenuates pro-inflammatory milieu by inhibiting nuclear factor (NF)-κB in *in vivo* and *in vitro* systems (31,41). In addition, MFG-E8 has recently been reported to have growth-promoting functions, where it promotes intestinal epithelial cell regeneration through the PKCε-mediated pathway (42). Furthermore, MFG-E8 is also known to promote AKT and Twist-dependent malignant melanoma progression (43) and ERK-mediated sperm-egg interaction (44), indicating its roles in manipulating intracellular signaling required for cell proliferation and their interaction with each other. Although the involvement of MAP kinases in controlling neutrophil migration has been well elucidated, to the best of our knowledge, there is no published study to date utilizing MFG-E8 for the activation of MAP kinases, which are linked to neutrophil migration. In this study, indicative of its role in immune cell migration, we observed a decrease in neutrophil migration as a result of the increased phosphorylation of p38 and ERK MAP kinases, which led to the downregulation of CXCR2 surface expression through the GRK2-dependent pathway. However, a recent study suggested that the different components of MAP kinases have distinct regulatory roles in neutrophil migration (25); the authors revealed the ‘stop’ and ‘go’ signal in the context of regulating neutrophil migration utilizing ERK and p38 phosphorylation, respectively. Based on their findings, the activation of p38 promoted, whereas the phosphorylation of ERK inhibited neutrophil migration. In accordance with their findings, in this study, using rhMFG-E8, we also found similar results in the context of the upregulation of ERK phosphorylation which may inhibit neutrophil migration. However, the difference between their study and ours is that in our study, rhMFG-E8 mediated the upregulation of p38 phosphorylation, inhibiting neutrophil migration, while in their study, p38 phosphorylation promoted neutrophil migration. However, in our study we tried to exclude this divergence by utilizing the p38 inhibitor, SB203580, which diminished the rhMFG-E8-induced down-regulation of neutrophil migration, hence suggesting a negative regulatory role of p38 in IL-8-mediated neutrophil migration upon stimulation with rhMFG-E8. The study carried out by Liu *et al* is to some extent different from our study in that they utilized fMLP as a chemoattractant and revealed the regulatory mechanism of neutrophil migration by treating dHL-60 cells directly with fMLP, and although the phosphorylation of p38 and ERK was induced, the feedback inhibition was initiated only by phospho-ERK (25). The involvement of MAP kinases in neutrophil migration can also be explained by another study in which pre-treatment of neutrophils with TLR ligands attenuated neutrophil migration due to high GRK2 expression (45). Since TLR ligations to their ligands induces the activation of MAP kinases, it is therefore conceivable that due to the activation of MAP kinases, the migration of neutrophils may be attenuated, which is in agreement with our findings that the MFG-E8-mediated upregulation of MAP kinase phosphorylation attenuated neutrophil migration. MFG-E8 contains EGF domains at its N-terminal domain. Since several proteins which have an EGF domain in their backbone, e.g., EGF, heparin-binding EGF-like growth factor (HB-EGF), Notch, and growth arrest-specific 6 (Gas6) are known to upregulate MAP kinases to execute their relevant functions (46–49), the upregulation of MAP kinase phosphorylation by rhMFG-E8 may be comparable.

Developmental endothelial locus-1 (Del-1), a probable paralogue protein of MFG-E8, which has a sequence and domain structure similar to MFG-E8, also shows identical biochemical functions of divalent binding activity to cell membrane molecules, such as MFG-E8 (50,51). The RGD motif in the second EGF-like domain is conserved between MFG-E8 and Del-1, both of which show binding to cells expressing α_v_β_3_ and α_v_β_5_ integrins. A recent study utilizing Del-1 indicated a significant inhibition of neutrophil migration through the blocking of the interaction of leukocyte functional antigen-1 (LFA-1) iin neutrophils and intercellular adhesion molecule-1 (ICAM-1) in endothelial cells, hence inhibiting neutrophil migration (52). In this study, we revealed a mechanism involving MAP kinases and the GRK2-mediated downregulation of CXCR2 by stimulation with MFG-E8 for the inhibition of neutrophil migration; this provides a novel direction for the modulation of the intracellular signaling cascade. Since MFG-E8 and Del-1 are homologous, the findings regarding Del-1 may be implemented so as to reveal the complete mechanisms through which MFG-E8 attenuates neutrophil migration. In this regard, since MFG-E8 has RGD in its backbone, emphasis therefore be placed on whether MFG-E8 blocks the interaction of extracellular matrix proteins to their integrin receptor to attenuate their binding for the initial attachment of neutrophils to endothelial cells as the first step of neutrophil migration. Therefore, deducing the role of MFG-E8 to the steps of roling and adherence may prove to be of considerable interest.

In conclusion, in this study, we identified a novel link between MAP kinases and GRK2, playing a negative regulatory role on CXCR2 surface expression (Fig. 6C). Our data may lead to translational studies being carried out for the identification of potential drug candidates which can modulate neutrophil migration, leading to the remission of several inflammatory diseases in which controlling exaggerated neutrophil infiltration is a major challenge.

## Abbreviations

MFG-E8: milk fat globule-epidermal growth factor-factor 8
NF-κB: nuclear factor-κB
MAP kinase: mitogen-activated protein kinase
ROS: reactive oxygen species
ALI: acute lung injury
BALF: bronchoalveolar lavage fluid
ARDS: acute respiratory distress syndrome
GRK2: G protein-coupled receptor kinase 2
GPCR: G protein-coupled receptor
PMNs: polymorphonuclear leukocytes

## References

[1] Nathan C (2006). Neutrophils and immunity: Challenges and opportunities. Nat Rev Immunol.

[2] Rot A, von Andrian UH (2004). Chemokines in innate and adaptive host defense: basic chemokinese grammar for immune cells. Annu Rev Immunol.

[3] Etzioni A, Tonetti M (2000). Leukocyte adhesion deficiency II-from A to almost Z. Immunol Rev.

[4] Webb PR, Wang KQ, Scheel-Toellner D, Pongracz J, Salmon M, Lord JM (2000). Regulation of neutrophil apoptosis: a role for protein kinase C and phosphatidylinositol-3-kinase. Apoptosis.

[5] Ayala A, Chung CS, Lomas JL, Song GY, Doughty LA, Gregory SH, Cioffi WG, LeBlanc BW, Reichner J, Simms HH, Grutkoski PS (2002). Shock-induced neutrophil mediated priming for acute lung injury in mice: divergent effects of TLR-4 and TLR-4/FasL deficiency. Am J Pathol.

[6] Abraham E (2003). Neutrophils and acute lung injury. Crit Care Med.

[7] Aziz M, Jacob A, Yang W-L, Matsuda A, Wang P (2013). Current trends in inflammatory and immunomodulatory mediators in sepsis. J Leukoc Biol.

[8] Aziz M, Matsuda A, Yang W-L, Jacob A, Wang P (2012). Milk fat globule-epidermal growth factor-factor 8 attenuates neutrophil infiltration in acute lung injury via modulation of CXCR2. J Immunol.

[9] Cui T, Miksa M, Wu R, Komura H, Zhou M, Dong W, Wang Z, Higuchi S, Chaung W, Blau SA (2010). Milk fat globule epidermal growth factor 8 attenuates acute lung injury in mice after intestinal ischemia and reperfusion. Am J Respir Crit Care Med.

[10] Wagner JG, Roth RA (2000). Neutrophil migration mechanisms, with an emphasis on the pulmonary vasculature. Pharmacol Rev.

[11] Lee WL, Downey GP (2001). Neutrophil activation and acute lung injury. Curr Opin Crit Care.

[12] Zarbock A, Ley K (2008). Mechanisms and consequences of neutrophil interaction with the endothelium. Am J Pathol.

[13] Donnelly SC, Strieter RM, Kunkel SL, Walz A, Robertson CR, Carter DC, Grant IS, Pollok AJ, Haslett C (1993). Interleukin-8 and development of adult respiratory distress syndrome in at-risk patient groups. Lancet.

[14] Goodman RB, Strieter RM, Martin DP, Steinberg KP, Milberg JA, Maunder RJ, Kunkel SL, Walz A, Hudson LD, Martin TR (1996). Inflammatory cytokines in patients with persistence of the acute respiratory distress syndrome. Am J Respir Crit Care Med.

[15] Olson TS, Ley K (2002). Chemokines and chemokine receptors in leukocyte trafficking. Am J Physiol Regul Integr Comp Physiol.

[16] Reutershan J, Morris MA, Burcin TL, Smith DF, Chang D, Saprito MS, Ley K (2006). Critical role of endothelial CXCR2 in LPS-induced neutrophil migration into the lung. J Clin Invest.

[17] Murphy PM (1997). Neutrophil receptors for interleukin-8 and related CXC chemokines. Semin Hematol.

[18] Johnston RA, Mizgerd JP, Shore SA (2005). CXCR2 is essential for maximal neutrophil recruitment and methacholine responsiveness after ozone exposure. Am J Physiol Lung Cell Mol Physiol.

[19] Vroon A, Heijnen CJ, Kavelaars A (2006). GRKs and arrestins: Regulators of migration and inflammation. J Leukoc Biol.

[20] Chuang TT, Iacovelli L, Sallese M, De Blasi A (1996). G protein- coupled receptors: Heterologous regulation of homologous desensitization and its implications. Trends Pharmacol Sci.

[21] Aragay AM, Ruiz-Gómez A, Penela P, Sarnago S, Elorza A, Jiménez-Sainz MC, Mayor F (1998). G protein-coupled receptor kinase 2 (GRK2): mechanisms of regulation and physiological functions. FEBS Lett.

[22] Penn RB, Pronin AN, Benovic JL (2000). Regulation of G protein-coupled receptor kinases. Trends Cardiovasc Med.

[23] Penela P, Ribas C, Mayor F (2003). Mechanisms of regulation of the expression and function of G protein-coupled receptor kinases. Cell Signal.

[24] Pitcher JA, Tesmer JJ, Freeman JL, Capel WD, Stone WC, Lefkowitz RJ (1999). Feedback inhibition of G protein-coupled receptor kinase 2 (GRK2) activity by extracellular signal-regulated kinases. J Biol Chem.

[25] Liu X, Ma B, Malik AB, Tang H, Yang T, Sun B, Wang G, Minshall RD, Li Y, Zhao Y (2012). Bidirectional regulation of neutrophil migration by mitogen-activated protein kinases. Nat Immunol.

[26] Hanayama R, Tanaka M, Miwa K, Shinohara A, Iwamatsu A, Nagata S (2002). Identification of a factor that links apoptotic cells to phagocytes. Nature.

[27] Hanayama R, Tanaka M, Miyasaka K, Aozasa K, Koike M, Uchiyama Y, Nagata S (2004). Autoimmune disease and impaired uptake of apoptotic cells in MFG-E8-deficient mice. Science.

[28] Ensslin MA, Shur BD (2003). Identification of mouse sperm SED1, a bimotif EGF repeat and discoidin-domain protein involved in sperm-egg binding. Cell.

[29] Hanayama R, Nagata S (2005). Impaired involution of mammary glands in the absence of milk fat globule EGF factor 8. Proc Natl Acad Sci USA.

[30] Ensslin MA, Shur BD (2007). The EGF repeat and discoidin domain protein, SED1/MFG-E8, is required for mammary gland branching morphogenesis. Proc Natl Acad Sci USA.

[31] Aziz M, Jacob A, Matsuda A, Wu R, Zhou M, Dong W, Yang W-L, Wang P (2011). Pre-treatment of recombinant mouse MFG-E8 downregulates LPS-induced TNF-α production in macrophages via STAT3-mediated SOCS3 activation. PLoS One.

[32] Brissette MJ, Lepage S, Lamonde AS, Sirois I, Groleau J, Laurin LP, Cailhier JF (2012). MFG-E8 released by apoptotic endothelial cells triggers anti-inflammatory macrophage reprogramming. PLoS One.

[33] Matsuda A, Jacob A, Wu R, Zhou M, Nicastro JM, Coppa GF, Wang P (2011). Milk fat globule-EGF factor VIII in sepsis and ischemia-reperfusion injury. Mol Med.

[34] Nuzzi PA, Lokuta MA, Huttenlocher A (2007). Analysis of neutrophil chemotaxis. Methods Mol Biol.

[35] Qiang X, Li J, Wu R, Ji Y, Chaung W, Dong W, Wang P (2011). Expression and characterization of recombinant human milk fat globule-EGF factor VIII. Int J Mol Med.

[36] Schneider CA, Rasband WS, Eliceiri KW (2012). NIH Image to ImageJ: 25 years of image analysis. Nat Methods.

[37] Belperio JA, Keane MP, Burdick MD, Londhe V, Xue YY, Li K, Phillips RJ, Strieter RM (2002). Critical role for CXCR2 and CXCR2 ligands during the pathogenesis of ventilator-induced lung injury. J Clin Invest.

[38] Matsuda A, Wu R, Jacob A, Komura H, Zhou M, Wang Z, Aziz MM, Wang P (2011). Protective effect of milk fat globule-epidermal growth factor-factor VIII after renal ischemia-reperfusion injury in mice. Crit Care Med.

[39] Brooks PC, Clark RA, Cheresh DA (1994). Requirement of vascular integrin alpha v beta 3 for angiogenesis. Science.

[40] Cheyuo C, Jacob A, Wu R, Zhou M, Qi L, Dong W, Ji Y, Chaung WW, Wang H, Nicastro J (2012). Recombinant human MFG-E8 attenuates cerebral ischemic injury: Its role in anti-inflammation and anti-apoptosis. Neuropharmacology.

[41] Aziz MM, Ishihara S, Mishima Y, Oshima N, Moriyama I, Yuki T, Kadowaki Y, Rumi MA, Amano Y, Kinoshita Y (2009). MFG-E8 attenuates intestinal inflammation in murine experimental colitis by modulating osteopontin-dependent alphavbeta3 integrin signaling. J Immunol.

[42] Bu HF, Zuo XL, Wang X, Ensslin MA, Koti V, Hsueh W, Raymond AS, Shur BD, Tan XD (2007). Milk fat globule-EGF factor 8/lactadherin plays a crucial role in maintenance and repair of murine intestinal epithelium. J Clin Invest.

[43] Jinushi M, Nakazaki Y, Carrasco DR, Draganov D, Souders N, Johnson M, Mihm MC, Dranoff G (2008). Milk fat globule EGF-8 promotes melanoma progression through coordinated Akt and twist signaling in the tumor microenvironment. Cancer Res.

[44] Raymond A, Ensslin MA, Shur BD (2009). SED1/MFG-E8: A bi-motif protein that orchestrates diverse cellular interactions. J Cell Biochem.

[45] Alves-Filho JC, Sônego F, Souto FO, Freitas A, Verri WA, Auxiliadora-Martins M, Basile-Filho A, McKenzie AN, Xu D, Cunha FQ (2010). Interleukin-33 attenuates sepsis by enhancing neutrophil influx to the site of infection. Nat Med.

[46] Oda K, Matsuoka Y, Funahashi A, Kitano H (2005). A comprehensive pathway map of epidermal growth factor receptor signaling. Mol Syst Biol.

[47] Reynolds CM, Eguchi S, Frank GD, Motley ED (2002). Signaling mechanisms of heparin-binding epidermal growth factor-like growth factor in vascular smooth muscle cells. Hypertension.

[48] Liu ZJ, Xiao M, Balint K, Smalley KS, Brafford P, Qiu R, Pinnix CC, Li X, Herlyn M (2006). Notch1 signaling promotes primary melanoma progression by activating mitogen-activated protein kinase/phosphatidylinositol 3-kinase-Akt pathways and up-regulating N-cadherin expression. Cancer Res.

[49] Goruppi S, Ruaro E, Schneider C (1996). Gas6, the ligand of Axl tyrosine kinase receptor, has mitogenic and survival activities for serum starved NIH3T3 fibroblasts. Oncogene.

[50] Hidai C, Zupancic T, Penta K, Mikhail A, Kawana M, Quertermous EE, Aoka Y, Fukagawa M, Matsui Y, Platika D (1998). Cloning and characterization of developmental endothelial locus-1: an embryonic endothelial cell protein that binds the alphavbeta3 integrin receptor. Genes Dev.

[51] Hanayama R, Tanaka M, Miwa K, Nagata S (2004). Expression of developmental endothelial locus-1 in a subset of macrophages for engulfment of apoptotic cells. J Immunol.

[52] Choi EY, Chavakis E, Czabanka MA, Langer HF, Fraemohs L, Economopoulou M, Kundu RK, Orlandi A, Zheng YY, Prieto DA (2008). Del-1, an endogenous leukocyte-endothelial adhesion inhibitor, limits inflammatory cell recruitment. Science.

